# Oral health related quality of life in a group of Egyptian preschool children with early childhood caries: a cross-sectional study

**DOI:** 10.1186/s12903-026-07655-6

**Published:** 2026-02-09

**Authors:** Sara Magdy, Asmaa Abou Bakr, Sara A. Botros, Ola Abd El Geleel

**Affiliations:** 1 Paediatric Dentistry and Dental Public Health Department, Faculty of Dentistry, The British University in Egypt, El Sherouk City, Egypt; 2 Oral Medicine and Periodontology, Faculty of Dentistry, Galala University, Suez, Egypt; 3 Conservative and Esthetic Dentistry Department, Faculty of Dentistry, The British University in Egypt, El Sherouk City, Egypt; 4 Pediatric Dentistry Department and Dental Public Health Department, Faculty of Dentistry, Ain Shams University, Cairo, Egypt

**Keywords:** Quality of life, Preschool children, Early childhood caries, ECOHIS

## Abstract

**Background:**

Early childhood caries (ECC) is a common dental condition that could affect preschool children ‘s functional and emotional well-being worldwide. Therefore, this study aimed to evaluate the impact of ECC on preschool Egyptian children’s Oral Health Related Quality of Life (OHRQoL) and their parents.

**Materials and methods:**

This observational cross-sectional study included 260 preschool children aged 3 to 5 years who attended the Pediatric Outpatient Clinic at Ain Shams University. A resident dentist performed clinical examinations to measure the dmft index (decayed, missing, and filled teeth) using dental examination tools following the World Health Organization (WHO) criteria for diagnosing dental caries. Caries-free children were also included to enable direct comparison and estimate the impact of ECC severity on quality of life. The Arabic version of the Early Childhood Oral Health Impact Scale (A-ECOHIS) was used in this study to evaluate their OHRQoL. Data from the questionnaire were tabulated, summarized, and statistically analysed using Shapiro–Wilk, Mann–Whitney U and, the Kruskal–Wallis tests. Correlations assessments were made using Spearman’s rank correlation.

**Results:**

Out of 260 children who participated in the study, 75.8% had ECC, of whom 31% had ECC and 69% had severe ECC (S-ECC). The mean ECOHIS score was 19.52 ± 12.46. Children with S-ECC had a significantly higher mean ECOHIS score (25.87 ± 10.05) than children without caries (4.94 ± 6.08) and those with ECC (20.35 ± 8.96) (*p* < 0.0001). Regression analysis verified that worse OHRQoL was significantly associated with the presence of ECC and rising dmft scores. Higher ECOHIS scores were also associated with maternal employment and parental age < 35 years.

**Conclusions:**

Lower OHRQoL was significantly associated with the high prevalence of ECC and severe ECC (S-ECC), especially in domains related to pain, eating difficulties, and psychological discomfort. Higher dmft scores, the presence of ECC, younger parental age, and employment were also associated with worse OHRQoL scores. These results emphasize the importance of early preventive dental care, particularly for preschool children in resource-limited settings.

**Supplementary Information:**

The online version contains supplementary material available at 10.1186/s12903-026-07655-6.

## Background

A person’s perceptions of their position in life according to their culture, goals, expectations, standards, and priorities” is how the WHO defines quality of life [1]. Correspondingly, it is dependent on an individual’s perception of various facets of life, which is subjective and invisible to others. Consequently, each person’s conditional traits and social, cultural, and environmental status have an impact on their quality of life.Children, unlike adults, are not able to fully express their feelings towards oral health, and their needs, adding to this is their imagination. As children grow up, their oral health becomes influenced by many factors, including their families, society, and their peers. Among the family factors that affect their OHRQoL are the socioeconomic status, education level, and their beliefs [2]. Children are susceptible to a range of oral conditions that may impair their quality of life, functionality, and general health. OHRQoL is also impacted by children’s oral health issues and habits [3].

Preschoolers’ linguistic and cognitive development present challenges for the OHRQoL tool’s development [4, 5]. Furthermore, childhood is full of transformations. Dental and facial features can also change quickly, in addition to psychosocial awareness and physiological development, necessitating a more suitable theoretical and conceptual framework for the development of the OHRQoL tool.Seven tools were published between 2006 and 2017, which were tailored for application in preschool children only for assessing the HRQoL. Among these tools is ໿the Early Childhood Oral Health Impact Scale (ECOHIS), which is the most used scale worldwide [6, 7].

The prevalence of dental caries among Egyptian children was higher in primary dentition (dmft and deft) when compared to permanent dentition (DMFT) [8] this is similar to what has been reported in India. ECC is a severe dental condition affecting many preschool children around the world. Dental caries affects children’s oral and general well-being throughout their lives [9]. Children suffering from ECC experience pain when drinking hot or cold liquids, difficulty biting and chewing, decreased appetite, weight loss, trouble sleeping, in addition to behavioural changes like impatience and low self-esteem, and a decline in academic performance [10]. An estimated 60% to 90% of school-age children are thought to have dental caries [11, 12]. This percentage varies significantly among populations, with dental caries incidence being significantly greater in underdeveloped nations, including the Middle East, than in wealthy nations [13]. In Egypt, 74% of school-age children of school age suffered from dental caries [8, 14].

The effect of untreated dental caries on preschoolers’ oral health-related quality of life (OHRQoL) has been examined in earlier research [15–18] but little research has been done expressly on the DMFT index and its association with the ECOHIS in Egyptian preschoolers. Oral diseases have a major impact on speech, nutrition, self-esteem, and general quality of life, the link between oral health and overall well-being is not well understood by the general Egyptian population. The systemic effects of oral health problems are often underestimated by people, which causes care to be delayed and results in deterioration [19–21]. Therefore, increasing public knowledge of the link between oral and general health is essential to encourage prompt prevention, early intervention, and better overall health outcomes for our population [22, 23].

Early Childhood Caries (ECC) continues to be one of the most common chronic diseases affecting preschool children in the world today and has serious effects on their overall and oral health. ECC clinical impacts, pain, feeding, and sleep problems are known, yet its psychosocial consequences on the child and the family are not well studied and documented, mainly in underdeveloped countries as Egypt. Oral Health-Related Quality of Life (OHRQoL) provides a patient-centered measure to evaluate functional, emotional, and social consequences of oral conditions. Early Childhood Oral Health Impact Scale (ECOHIS) has been cross-culturally validated and translated into Arabic; and it constitutes a dependable instrument for OHRQoL assessment in young children. The null hypothesis is that there is no association between EEC and the ECOHIS scores in Egyptian preschool children. And since few studies associating ECC with ECOHIS scores have been conducted on Egyptian preschool children, we sought to address this gap by examining the relationship between ECC and OHRQoL in this group and thus adding to the available evidence from other parts of the world.

##  Subjects and methods

### Sample size calculation

The size of the target population of preschool children aged from 3 to 5 years old presenting yearly at the Paediatric Dentistry Department Outpatient Clinics at Faculty of Dentistry, Ain Shams University was estimated to be 6000 children. The sample size was calculated using the sample size calculator by Raosoft, Inc. (http://www.raosoft.com/samplesize.html) with a 5% margin of error, a 95% confidence interval, and an 80% estimated response rate. The minimal sample size needed was *N* = 237 children, and in compensation for any anticipated losses, the sample size was increased by 10% and adjusted to *N* = 260 children [24].

Study design and setting This research was designed as a cross-sectional observational study, which was carried out in the Department of Pediatric Dentistry and Dental Public Health, Faculty of Dentistry, Ain Shams University, and The British University in Egypt. The study adhered to the ‘Strengthening the Reporting of Observational studies in Epidemiology’ (STROBE) guidelines as outlined in Supplemental Material 1.

Study timeline The study was conducted at the Pediatric Dentistry Outpatient Clinic, Ain Shams University, and The British University in Egypt over 7 months (November 2024– May 2025).

### Ethical considerations

The current study was conducted following the Helsinki Declaration. The study protocol was reviewed and approved by the Ain Shams research ethics committee and was given a reference number (FDASU-Rec IR102424). The purpose of the study was explained to the parents of eligible children, and before participation, the parents were asked to sign an “Informed Consent Form”.

### Eligibility criteria

**Fig. 1:**
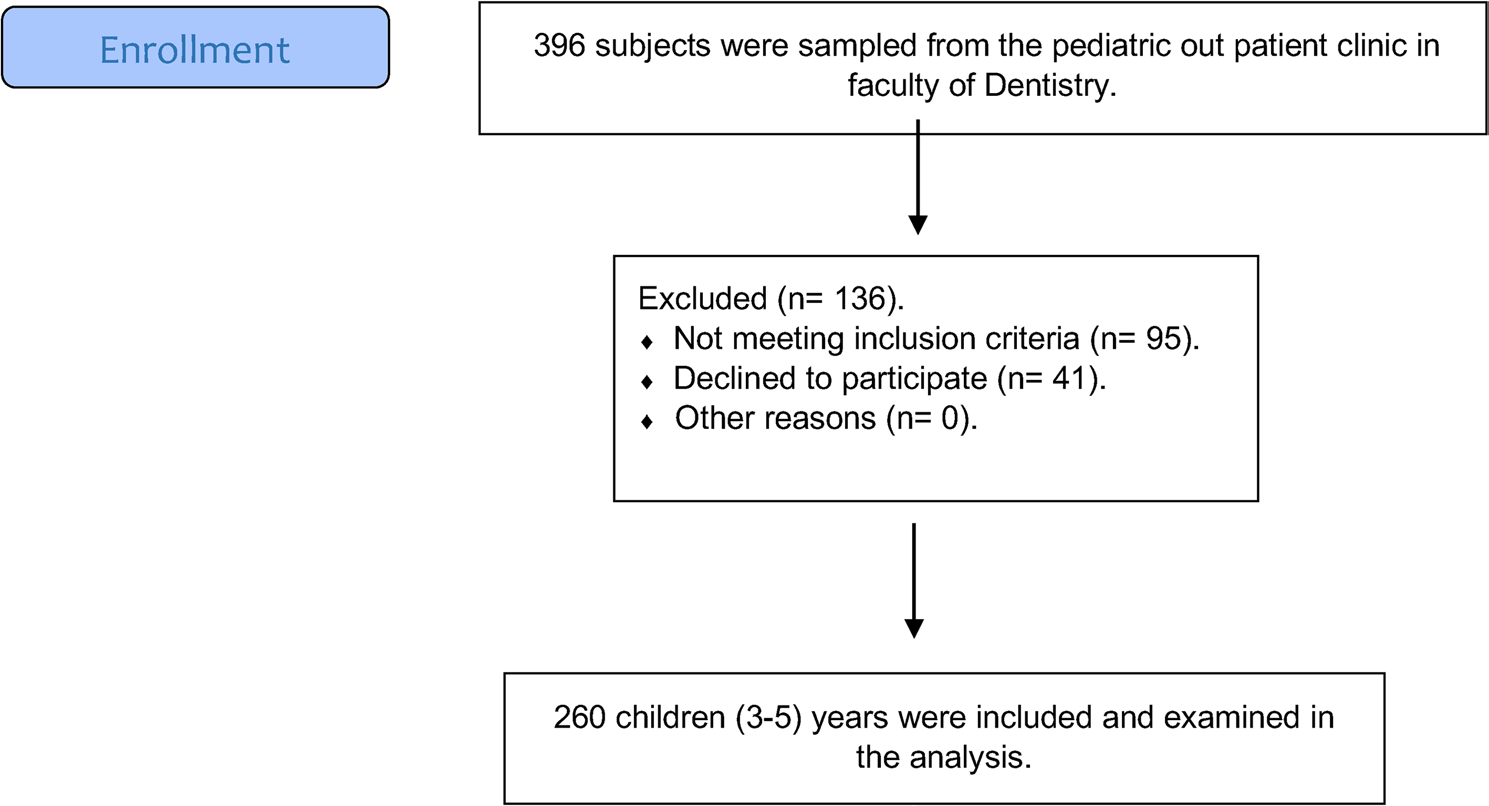
Flow diagram for patients’ recruitment

Preschool Children with an age range from 3 to 5 years old were included in the study, while those who were medically compromised, on prescription, or children whom their parents refused to sign an informed consent were also excluded from the study sample without any implications on the services utilized by those patients. Patients’ enrolment is illustrated in Fig. 1.

Preschool Children with an age range from 3 to 5 years old were included in the study, while those who were medically compromised (children with physical, mental, developmental, sensory, behavioral, cognitive, or emotional impairments that can affect their health), or children whom their parents refused to sign an informed consent were also excluded from the study sample without any implications on the services utilized by those patients [7, 25]. The exclusion of the medically compromised children was implemented to minimize the confounding factors, as the complex health needs and higher risk for oral diseases in such children could significantly alter their oral health-related quality of life scores independent of the common oral conditions under investigation, thereby threatening the internal validity of the study [26]. Moreover, these criteria ensured the homogeneity of the population. Patients’ enrolment is illustrated enrolment is illustrated in Fig. 1.

### Sampling strategy

Participants were chosen using a non-probability convenience sampling technique. Children between the ages of three and five who visited the Pediatric Dentistry Outpatient Clinics at Ain Shams University’s Faculty of Dentistry were considered for the study. Regardless of whether they were visiting for treatment, follow-up, or to accompany a sibling or family member receiving dental care, eligible participants were gathered one after the other throughout the study period. Recruitment proceeded until the desired sample size was reached, and participants were added following the predetermined eligibility criteria.

### Outcomes measures

The assessment of the OHRQoL using the Arabic version of the Early Childhood Oral Health Impact scale (A-ECOHIS) is the primary outcome. While the secondary outcomes include measuring ECC prevalence, and the identification of possible associations between sociodemographic and clinical predictors and the total ECOHIS scores.

### Study procedures

A demographic data questionnaire was filled out by the contributing parents, which included information on the child’s age, gender, and parental age and employment status.

Clinical examination was then carried out on all the participating children by a single pre-calibrated skilled pedodontist to guarantee consistency and avoid inter-examiner variability using dental examination tools (disposable dental mirror, dental explorer, sterile gauze, and mask) according to the WHO criteria for caries diagnosis [27]. The examining pedodontist participated in a calibration training session prior to the study’s start. Intra-examiner reliability of dmft scoring between the two assessment time points was excellent. Caries experience was evaluated utilizing the dmft index (decayed, missed, filled index) as it was suitable to use on the examined age group.

The presence of Early Childhood Caries (ECC) was identified by detecting one or more decayed, missing, or filled tooth surfaces due to caries in a child younger than 6 years, as provided by the AAPD. In addition, the presence of any smooth surface caries in children younger than 3 years is indicative S-ECC, while from the age of 3 through 5 years, the following scores are also considered S-ECC when the dmf score is greater than or equal to [4] in 3-year-olds, greater than or equal to [5] in 4-year-olds and greater than or equal to [6] in 5-year-olds [14, 28].

Afterwards, the parents of the children were asked to answer the Arabic version of the ECOHIS (A-ECOHIS) questionnaire while conducting in-person interviews by the same examiner/interviewer, as the obtained scores provide an evaluation of the quality of life related to oral health.

The questionnaire comprised 13 questions covered in two sections: the child impact section (CIS) and the family impact section (FIS). The first section includes inquiries about the Child’s symptoms (1 item), child functions (4 items), child psychology (2 items), and child self-image and social interaction (2 items), while the (FIS) includes other inquiries that represent the family function (2 items) and parental distress (2 items) [26].

The questions included in the questionnaire are based on a 4-point Likert scale, with answers ranging from never = 0 to very often = 4, as they denote how frequently an event is encountered. In cases where parents responded by “don’t know”, in one or more of the questions in the (FIS) or two or more in the (CIS), their questionnaires were eliminated from the analysis.

The (CIS) items’ total score falls between (0–36), while that of the (FIS) is between (0–16). Then the final score is obtained by adding the scores of the aforementioned sections, such that the final score ranges from 0 to 52. A child’s quality of life is negatively impacted by their oral health, as shown by a higher score [29].

### Statistical analysis

Statistical analyses were conducted using Stata version 18.0 (StataCorp LLC, College Station, TX, USA). Categorical variables were summarized as frequencies and percentages, and continuous variables as means ± standard deviations (SD). The Shapiro–Wilk test was used to assess normality. Given the non-normal distribution of ECOHIS scores, non-parametric tests were applied: the Mann–Whitney U test for two-group comparisons and the Kruskal–Wallis test for more than two groups, with Bonferroni-adjusted post hoc tests as needed. Spearman’s rank correlation assessed associations between ECOHIS scores and continuous variables, reporting correlation coefficients and 95% confidence intervals (CIs). Multivariable linear regression was used to identify predictors of the total ECOHIS score, reporting β coefficients, 95% CIs, and p-values. Additionally, binary logistic regression was applied to assess the association between predictor variables and the likelihood of domain-specific impacts, expressed as odds ratios (ORs) with 95% CIs. A two-sided p-value of < 0.05 was considered statistically significant.

## Results

### Participants’ demographics and clinical characteristics

A total of 260 children participated in the study, with a mean age of 4.33 ± 0.79 years. Nearly equal proportions were aged ≤ 4 years (49.6%) and > 4 years (50.4%). The sample included 125 males (48%) and 135 females (52%). Regarding dental caries experience, 75.8% of the children had early childhood caries (ECC), of whom 31.0% had ECC and 69.0% had severe ECC (S-ECC). The mean decayed, missing, and filled teeth (dmft) score was 5.38 ± 4.49, with mean values of 4.93 ± 4.29 for decayed teeth, 0.19 ± 0.59 for missing teeth, and 0.29 ± 0.70 for filled teeth. Among the children, 24.2% had a dmft score of 0, 26.2% had scores of 1–5, and 49.6% had scores > 5. The parents had a mean age of 31.92 ± 7.31 years, with 71.5% aged < 35 years. Most parents were mothers (93.1%), while 6.9% were fathers. In terms of employment, 46.2% of parents were employed and 53.8% were unemployed (Table 1).

**Table 1:** Demographic and Clinical Characteristics of the Study Sample (n = 260)

Variable	Frequency (%)
*Child’s demographics*:	
Age	
(Mean ± SD)	4.33 ± 0.79 years
≤ 4 y	129 (49.6%)
> 4 y	131 (50.4%)
Gender	
Male	125 (48%)
Female	135(52%)
Caries experience:	
ECC Incidence	
Present	197 (75.8%)
Absent	63 (24.2%)
ECC Severity	
ECC	61 (31.0%)
S-ECC	136 (69.0%)
dmft Score	
0	63 (24.2%)
1–5	68 (26.2%)
> 5	129 (49.6%)
d (Mean ± SD)	4.93 ± 4.29
m (Mean ± SD)	0.19 ± 0.59
f (Mean ± SD)	0.29 ± 0.70
Total dmf (Mean ± SD)	5.38 ± 4.49
*Parent’s demographics*:	
Age	
(Mean ± SD)	31.92 ± 7.31 years
< 35 y	186 (71.5%)
≥ 35 y	74 (28.5%)
Relationship to child	
Mother	242 (93.1%)
Father	18 (6.9%)
Employment status	
Employed	120 (46.2%)
Unemployed	140 (53.8%)

### ECOHIS response distribution patterns

Parental responses to the ECOHIS revealed that a considerable proportion of children experienced oral health-related impacts “often” or “very often.” Tooth pain was the most frequently reported issue, affecting 48.1% of children (25.4% “often”, 22.7% “very often”), followed by difficulty tolerating hot/cold drinks (44.3%), eating difficulties (46.9%), and school absenteeism (29.3%). Sleep disturbances (33.4%) and irritability (33.1%) were also common. Smiling difficulty and speaking difficulty were less frequently reported, at 13.9% and 10.8%, respectively. Regarding family impacts, 37.7% of parents reported feeling angry given their children’s oral health status, 33.1% reported feelings of guilt, and 10.0% reported missing workdays. Remarkably, 68.0% of respondents indicated that their child’s oral health condition had a financial impact on the family. These findings highlight both the functional and emotional burden of early childhood caries on affected children and their families (Table 2).

**Table 2:** Distribution of Responses to the Early Childhood Oral Health Impact Scale (ECOHIS) items (n = 260)

	Never	Hardly ever	Occasionally	Often	Very often	Don’t know
Impact on the child						
1. Painful teeth	54 (20.8%)	23 (8.8%)	57 (21.9%)	66 (25.4%)	59 (22.7%)	0 (0%)
2. Tolerating hot/cold drinks	61 (23.5%)	36 (13.8%)	47 (18.1%)	68 (26.2%)	47 (18.1%)	2 (0.8%)
3. Eating difficulty	70 (26.9%)	27 (10.4%)	41 (15.8%)	77 (29.6%)	45 (17.3%)	0 (0%)
4. Pronouncing words difficulty	160 (61.5%)	32 (12.3%)	18 (6.9%)	32 (12.3%)	14 (5.4%)	4 (1.5%)
5. Scholar absenteeism	102 (39.2%)	38 (14.6%)	46 (17.7%)	47 (18.1%)	29 (11.2%)	0 (0%)
6. Sleep disorder	82 (31.5%)	31 (11.9%)	57 (21.9%)	56 (21.5%)	31 (11.9%)	4 (1.5%)
7. Irritability and frustration	81 (31.2%)	41 (15.8%)	47 (18.1%)	45 (17.3%)	41 (15.8%)	5 (1.9%)
8. Smiling difficulty	162 (62.3%)	41 (15.8%)	20 (7.7%)	20 (7.7%)	16 (6.2%)	2 (0.8%)
9. Speaking difficulty	173 (66.5%)	38 (14.6%)	22 (8.5%)	14 (5.4%)	14 (5.4%)	0 (0%)
Impact on the family						
10. Feeling upset	86 (33.1%)	27 (10.4%)	48 (18.5%)	74 (28.5%)	24 (9.2%)	2 (0.8%)
11. Guilty feeling	111 (42.7%)	27 (10.4%)	36 (13.8%)	61 (23.5%)	25 (9.6%)	0 (0%)
12. Taken time of work	167 (64.2%)	20 (7.7%)	43 (16.5%)	22 (8.5%)	4 (1.5%)	5 (1.9%)
13. Financial impact	34 (13.1%)	13 (5.0%)	14 (5.4%)	153 (58.8%)	24 (9.2%)	24 (9.2%)

### Comparison of OHRQoL (ECOHIS) by ECC severity

Comparison of ECOHIS domain scores across caries-free, ECC, and S-ECC groups revealed statistically significant differences in all domains (*p* < 0.05), except for the psychological and social interaction domains, where ECC and S-ECC groups did not differ significantly. Children with S-ECC exhibited the highest total ECOHIS scores (25.87 ± 10.05), followed by those with ECC (20.35 ± 8.96), and caries-free children (4.94 ± 6.08), with all pairwise comparisons showing significant differences (*p* < 0.0001). Similarly, scores for the child function domain showed a progressive increase from caries-free (1.37 ± 2.20) to ECC (6.47 ± 3.92) to S-ECC (8.54 ± 3.81), with significant pairwise differences between all groups (*p* < 0.0001). For the child symptom and psychological domains, both ECC and S-ECC groups scored significantly higher than caries-free children (*p* < 0.0001) but did not differ significantly from one another (*p* = 0.0983 and 0.1634, respectively).

Notably, in the child self-image/social interaction domain, S-ECC children reported significantly higher scores (2.03 ± 2.68) than both caries-free (0.80 ± 1.47) and ECC children (0.97 ± 2.05), with a significant difference observed only between S-ECC and the other groups (*p* = 0.017). Regarding parental impact, both the distress and family function domains revealed significantly elevated scores in ECC and S-ECC groups compared to caries-free children (*p* < 0.0001), with the S-ECC group reporting higher parental distress than the ECC group (*p* = 0.0071), while no difference was found in the family function domain (*p* = 0.9125). These findings underscore the significant burden of early childhood caries on both the child’s quality of life and the family’s psychosocial well-being, with severity of caries directly associated with higher ECOHIS scores across multiple domains as presented in Table 3.

**Table 3:** Comparison of Mean ECOHIS Domain Scores Among Caries-Free, ECC, and S-ECC Children

Domain	Caries-free (*n* = 63)	ECC (*n* = 61)	S-ECC (*n* = 136)	*p*-value
I) Child impact:				
Symptoms	0.34 (0.68)ᵃ	2.53 (1.16)ᵇ	2.92 (0.98)ᵇ	< 0.0001*
Function	1.37 (2.20)ᵃ	6.47 (3.92)ᵇ	8.54 (3.81)ᶜ	< 0.0001*
Psychology	0.74 (1.34)ᵃ	3.71 (2.60)ᵇ	4.43 (2.50)ᵇ	< 0.0001*
Social interaction	0.80 (1.47)ᵃ	0.97 (2.05)ᵃ	2.03 (2.68)ᵇ	0.017*
II) Parent impact:				
Parental distress	0.51 (0.89)ᵃ	3.12 (2.20)ᵇ	4.38 (2.32)ᶜ	< 0.0001*
Family function	1.17 (1.48)ᵃ	3.56 (1.40)ᵇ	3.57 (1.81)ᵇ	< 0.0001*
III) Total ECOHIS	4.94 (6.08)ᵃ	20.35 (8.96)ᵇ	25.87 (10.05)ᶜ	< 0.0001*

Values are presented as mean (standard deviation). Superscripts (ᵃ, ᵇ, ᶜ) indicate statistically significant differences between groups based on post hoc pairwise comparisons following the Kruskal–Wallis test

Groups sharing the same superscript letter do not differ significantly at *p* < 0.05

### Association and predictors of OHRQoL

Spearman’s rank correlation analysis demonstrated several significant associations with total ECOHIS scores. Notably, pain presence showed the strongest positive correlation (ρ = 0.66; 95% CI: 0.56–0.74; *p* < 0.001), followed by ECC severity (ρ = 0.52; 95% CI: 0.40–0.63; *p* < 0.001) and dmft score (ρ = 0.49; 95% CI: 0.35–0.61; *p* < 0.001). A weaker but statistically significant positive correlation was observed with child age (ρ = 0.21; 95% CI: 0.04–0.37; *p* = 0.013). In contrast, parents’ age showed a non-significant trend (ρ = 0.13; 95% CI: − 0.03–0.29; *p* = 0.128). No significant correlations were found between total ECOHIS scores and gender (ρ = 0.02; 95% CI: − 0.13–0.17; *p* = 0.781), parents’ gender (ρ = 0.01; 95% CI: − 0.15–0.16; *p* = 0.911), parents’ age < 35 years (ρ = − 0.06; 95% CI: − 0.20–0.09; *p* = 0.420), or employment status (ρ = − 0.03; 95% CI: − 0.18–0.12; *p* = 0.715).

### Regression models

Following that, a multiple linear regression model was conducted to evaluate the relationship between sociodemographic and clinical predictors and total ECOHIS scores. The presence of ECC was significantly associated with higher total ECOHIS scores, with a coefficient of β = 12.22 (95% CI: 7.35 to 17.10; *p* < 0.0001). Additionally, the dmft score was positively associated with ECOHIS scores (β = 0.62; 95% CI: 0.05 to 1.20; *p* = 0.034), indicating that each unit increase in dmft was linked to a 0.62-point increase in ECOHIS score.

Other predictors, including child age (β = 1.43; 95% CI: − 0.61 to 3.47; *p* = 0.168), sex (female) (β = 0.412; 95% CI: − 2.64 to 3.46; *p* = 0.790), S-ECC status (vs. ECC) (β = 2.87; 95% CI: − 1.88 to 7.61; *p* = 0.2347), parent age < 35 years (β = − 0.34; 95% CI: − 3.21 to 2.53; *p* = 0.813), and the parents being the mother (β = − 0.344; 95% CI: − 3.214 to 2.527; *p* = 0.8132) were not statistically significant. Employment status returned a β coefficient of 0.000 with a fixed confidence interval [0.000, 0.000] and no p-value, likely due to complete separation or collinearity (Table 4).

**Table 4:** Multiple Linear Regression Analysis for Predictors of Total ECOHIS Score

Predictor	Coefficient (β)	95% CI	*p*-value
Child Age	1.43	[−0.60, 3.47]	0.168
Sex (Female)	0.41	[−2.64, 3.46]	0.790
ECC Present	**12.22**	[7.35, 17.10]	< 0.0001*
S-ECC (vs. ECC)	2.87	[−1.89, 7.61]	0.235
dmft Score	**0.62**	[0.05, 1.20]	0.034*
Parent < 35y	−0.34	[−3.21, 2.53]	0.813
Mother	−0.34	[−3.21, 2.53]	0.813
Employed	0.0	[0.00, 0.00]	—

*Statistically significant at p < 0.05

*ECC* Early Childhood Caries, *S-ECC* Severe ECC

Multivariable logistic regression analysis revealed that ECC presence was a significant predictor of increased adverse scores across all ECOHIS domains. In the *Child Impact* domain, children with ECC had 10.99 times higher odds of adverse impact compared to caries-free children (95% CI: 2.47–48.96; *p* = 0.002). Additionally, parents aged < 35 years were significantly associated with worse child impact outcomes (OR = 3.15; 95% CI: 1.28–7.76; *p* = 0.013). In the *Child Symptom* domain, ECC was associated with 83.36 times higher odds of adverse symptoms (95% CI: 14.33–484.88; *p* < 0.0001), and parents’ age < 35 years remained a significant predictor (OR = 12.31; 95% CI: 4.06–37.34; *p* < 0.0001). Employment status also showed a strong association (OR = 6.44; 95% CI: 6.44–6.44; *p* < 0.0001), though this estimate likely reflects quasi-complete separation in the data. For the *Child Function* domain, ECC presence remained significant (OR = 9.65; 95% CI: 2.09–44.58; *p* = 0.004). Similarly, ECC was a significant predictor of adverse *Child Psychological* impact (OR = 12.42; 95% CI: 2.15–71.58; *p* = 0.005). Although none of the predictors reached statistical significance in the *Child Social* domain, the odds ratio for ECC was 3.65 (95% CI: 0.85–15.63; *p* = 0.082).

In the *Parent Impact* domain, ECC was again a strong predictor (OR = 33.77; 95% CI: 6.72–169.72; *p* < 0.0001), as was employment status (OR = 4.15; 95% CI: 1.68–10.27; *p* = 0.002). This trend was consistent in the *Parental Distress* domain, where ECC (OR = 34.45; 95% CI: 5.65–210.05; *p* < 0.0001) and employment (OR = 4.05; 95% CI: 1.55–10.63; *p* = 0.004) remained statistically significant. Finally, in the *Family Function* domain, ECC presence was the only significant predictor (OR = 110.77; 95% CI: 16.81–730.01; *p* < 0.0001) (Table 5).

**Table 5:** Multivariable Logistic Regression of Predictors for Adverse Scores in ECOHIS Domains

Domain		Age > 4y	Female	ECC Incidence	S-ECC	dmf score	Parent < 35y	Mother	Employed
Child impact	OR	1.23 [0.55–2.76] 0.615	0.91 [0.41–2.02] 0.821	10.99 [2.47–48.96] 0.002*	1.21 [0.39–3.74] 0.739	1.07 [0.93–1.24] 0.347	3.15 [1.28–7.76] 0.013*	0.75 [0.16–3.43] 0.711	1.86 [0.82–4.19] 0.135
	CI								
	P								
Child symptom	OR	2.50 [0.66–9.53] 0.179	1.04 [0.27–4.00] 0.959	83.36 [14.33–484.88] < 0.0001*	4.65 [0.57–37.89] 0.151	1.18 [0.81–1.71] 0.394	12.31 [4.06–37.34] < 0.0001*	0.00 [0.00–inf] 1.000	6.44 [6.44–6.44] < 0.0001*
	CI								
	P								
Child function	OR	1.23 [0.52–2.86] 0.639	0.49 [0.21–1.14] 0.099	9.65 [2.09–44.58] 0.004*	1.46 [0.45–4.78] 0.528	1.12 [0.94–1.33] 0.206	2.25 [0.89–5.73] 0.087	0.91 [0.19–4.38] 0.906	1.55 [0.67–3.61] 0.307
	CI								
	P								
Child psychology	OR	1.07 [0.46–2.47] 0.878	1.12 [0.49–2.55] 0.793	12.42 [2.15–71.58] 0.005*	0.78 [0.23–2.57] 0.679	1.18 [0.98–1.42] 0.073	1.81 [0.71–4.64] 0.214	0.22 [0.02–1.91] 0.169	2.03 [0.87–4.76] 0.103
	CI								
	P								
Child social	OR	0.35 [0.74–2.76] 0.615	−0.09 [0.23–1.90] 0.445	3.65 [0.85–15.63] 0.082	1.16 [0.31–4.37] 0.825	1.04 [0.87–1.25] 0.671	1.68 [0.64–4.40] 0.292	0.58 [0.11–2.91] 0.507	1.52 [0.62–3.75] 0.358
	CI								
	P								
Parent impact	OR	0.89 [0.38–2.12] 0.797	0.94 [0.41–2.17] 0.880	33.77 [6.72–169.72] < 0.0001*	1.81 [0.54–6.07] 0.340	0.96 [0.83–1.12] 0.626	0.51 [0.19–1.42] 0.198	1.02 [0.22–4.74] 0.978	4.15 [1.68–10.27] 0.002*
	CI								
	P								
Parental distress	OR	1.23 [0.50–3.05] 0.657	1.09 [0.45–2.64] 0.845	34.45 [5.65–210.05] < 0.0001*	2.33 [0.65–8.35] 0.193	1.02 [0.86–1.20] 0.858	0.56 [0.19–1.63] 0.285	1.36 [0.28–6.47] 0.702	4.05 [1.55–10.63] 0.004*
	CI								
	P								
Family function	OR	1.11 [0.38–3.20] 0.848	0.66 [0.23–1.90] 0.445	110.77 [16.81–730.01] < 0.0001*	0.45 [0.07–2.90] 0.400	0.98 [0.81–1.18] 0.814	0.85 [0.26–2.76] 0.782	0.84 [0.09–7.60] 0.878	1.34 [0.47–3.82] 0.586
	CI								
	P								

*Statistically significant at *p* < 0.05

¹Fixed OR due to separation or quasi-complete separation in data; interpret with caution

### Intra-examiner reliability

The quadratic weighted Cohen’s kappa was 0.93 (95% CI: 0.86–0.99), indicating very high agreement. Similarly, the intraclass correlation coefficient [ICC [1, 2] was 0.93 (95% CI: 0.84–0.98), confirming excellent reliability of repeated scoring by the same examiner as presented in (Table 6).

**Table 6:** Intra-examiner reliability of dmft scores at two assessment time points

Reliability Measure	Value	95% CI	Interpretation
Weighted Cohen’s Kappa (κ)	0.93	0.86–0.99	Excellent agreement
Intraclass Correlation Coefficient (ICC [1, 2])	0.93	0.84–0.98	Excellent reliability

ICC [1, 2] was calculated using a two-way random-effects model, absolute agreement, single measures. Confidence intervals for κ were estimated via bootstrap resampling (1,000 iterations)

## Discussion

Despite widespread preventive strategies, Early childhood caries (ECC) remains a persistent and serious public health concern affecting many preschool-aged children, particularly in underserved regions with limited access to preventive dental care and low oral health literacy [23, 30]. Beyond its clinical implications, ECC can severely impact a child’s overall well-being, leading to pain, nutritional difficulties, disrupted sleep, and behavioural disturbances [31]. These effects extend beyond the child, often causing emotional distress, financial burdens, and increased caregiving demands on families [32]. Assessing oral health-related quality of life (OHRQoL) offers critical insights into the multidimensional consequences of ECC on both children and their families. This study aimed to evaluate these impacts using a validated preschool OHRQoL instrument, with a specific focus on quantifying the real-world burden of ECC in an Egyptian setting.

The Early Childhood Oral Health Impact Scale (ECOHIS) was employed to assess oral health-related quality of life in children aged 3–6 years. This validated instrument specifically evaluates how oral and dental conditions affect both the child’s daily functioning and family dynamics. ECOHIS has demonstrated strong reliability in measuring the oral health impacts of various conditions, including medical disorders, traumatic dental injuries, malocclusion, and early childhood caries [32]. 

The findings of this study revealed a high prevalence of early childhood caries (ECC), affecting more than three-fourths of the preschool-aged participants, with severe early childhood caries (S-ECC) present in 73.6% of these children. While the elevated prevalence may be partly attributed to the convenience sampling method—recruiting participants from a dental healthcare facility— these findings corroborate a previous nationwide survey in Egypt, which reported analogous ECC rates [33]. This consistency is reinforced by several local studies documenting similar prevalence levels in preschool-aged children [34]. Furthermore, studies from other developing nations have reported comparable figures [35–38].

Alarmingly, the recorded mean dmf score was higher than the mean scores obtained in the previously mentioned national studies [33, 34, 39], with the “d” (decayed) component of the index contributed the most significantly to the total score. This highlights a concerning level of untreated dental caries, which could stem from several factors, including parental misconceptions about primary teeth being expendable until permanent dentition emerges, despite their critical role in a child’s growth and development. Additionally, financial barriers further restrict access to care [40], particularly in urban areas where dental services are available but often deemed unaffordable by parents [41].

The mean ECOHIS score in the study sample was 19.52 (± 12.46), which is higher than values reported in earlier studies by Jaggi et al. [16]and Kurt et. al [26]This discrepancy may be explained by the greater severity of dental caries observed in our cohort, evidenced by higher dmf scores and a larger proportion of cases meeting the criteria for severe early childhood caries (S-ECC), both of which are likely associated with more pronounced clinical symptoms and impacts on oral health-related quality of life.

Parental responses to the individual items of the ECOHIS revealed that pain, functional limitations (such as difficulty with chewing and tolerating hot/cold drinks), and psychological discomfort (such as irritability, sleeping disorders) were the most reported issues. These findings are biologically plausible and consistent with caries experience in particular. Parental distress was the domain with the highest scores in the FIS component, since parents commonly feel upset, guilty, and financially strained in cases with untreated or recurring diseases, especially when symptomatic. Similar findings were obtained by Aldrigui et al. [42] and Scarpelli et al. [43], who reported that dental caries, among other oro/dental complaints as traumatic dental injuries, malocclusions, and developmental enamel defects, represents the leading negative impact on the quality of life of preschool children. Also, Perazzo et al. [44] described a similar pattern despite using an alternate scale, which is the Scale of Oral Health Outcomes for 5-Year-Old Children (SOHO-5) that evaluates the self-perceptions of children rather than relying on parental reports.

To determine key factors associated with reduced quality of life, regression analysis was implemented, considering both patient- and parent-related variables. The findings revealed that the presence of early childhood caries (ECC) significantly impairs the quality of life for children and their parents, reflected as a substantial negative influence on nearly all domains of the scale and in turn the total score (coefficient of β = 12.222,95% CI: 7.348 to 17.096; *p* < 0.0001). Furthermore, a dose-dependent relationship was evident between caries severity (dmf index) and oral health-related quality of life, where higher disease burden consistently correlated with worse OHRQoL scores. This pattern has been corroborated across multiple studies, reinforcing the validity of these associations [15, 18].

Additionally, multivariate logistic regression revealed that younger parental age was significantly associated with higher child impact scores, suggesting that older parents may be more experienced in managing their children’s health issues and less overwhelmed by symptoms such as pain and functional limitations. Moreover, maternal employment emerged as a significant predictor of reduced quality of life in our sample, given that mothers were the primary accompanying parents. This may stem from the emotional distress experienced by working mothers, including feelings of guilt and frustration, as they might perceive themselves as unable to adequately attend to their children’s health needs [15, 45].

On the other hand, the present study found no significant association between the child’s age or gender and the total ECOHIS score. These results are consistent with the findings of Abanto et al. [45], Jaggi et al. [16], and Yang et al. [46]. However, previous studies have suggested that older children, due to their more advanced language skills, may better express and communicate oral health concerns to their parents, potentially leading to higher reported impacts on quality of life [15, 47]. Additionally, some studies [46, 48, 49] have reported gender-based differences, with girls experiencing greater dental anxiety and being more affected by the aesthetic consequences of dental caries, which may negatively influence their oral health-related quality of life.

This study has several strengths that enhance the reliability and relevance of its findings. A key advantage was the use of the Arabic version of the Early Childhood Oral Health Impact Scale (ECOHIS), a validated and culturally adapted tool that ensures accurate assessment of oral health-related quality of life (OHRQoL) in Egyptian preschoolers. Direct comparisons were made possible by the inclusion of a caries-free control group, which also improved the interpretation of how ECC affected OHRQoL. Furthermore, a thorough understanding of contributing factors is provided by the use of multivariate models that incorporate both clinical and parents-related predictors.

By highlighting the psychosocial rather than the widely acknowledged clinical consequences of early childhood caries (ECC) on children and their families, this research provides valuable insights for paediatric dentists. Additionally, it identifies key child and parent-related factors linked to poor OHRQoL, offering an opportunity to educate parents on the importance of timely dental care to improve overall well-being.

Despite its contributions, this study is subject to important methodological limitations. The cross-sectional nature of the design inherently prevents the establishment of causal relationships between ECC and quality of life, limiting the findings to the identification of associations. Future research should employ longitudinal designs to explore these potential causal links. Additionally, the recruitment of participants from a single public institution may limit the generalizability of the results, as geographic and socioeconomic factors could influence the outcomes. Furthermore, the parental responses are usually influenced by their subjective interpretation of their child’s experiences and recall inaccuracies, which could introduce measurement bias due to the potential discrepancy between the reported and the lived experience. Despite being a validated tool, ECOHIS might not accurately represent the viewpoint of the child. In addition, the regression models used were unable to take into consideration unmeasured confounding variables that could impact both caries outcomes and reported quality of life, such as parental oral health literacy, dental anxiety, or other psychosocial influences.

## Conclusions

This study emphasizes the profound impact of ECC on the oral health-related quality of life (OHRQoL) in a group of Egyptian preschool-aged children and their families, as measured by the validated ECOHIS tool. The high prevalence of ECC (affecting > 75% of participants) and severe ECC (73.6% of the affected children), coupled with elevated mean dmf scores, reflects a critical gap in preventive and restorative care. Key findings revealed that ECC significantly impairs OHRQoL across nearly all the scale domains, with pain, functional limitations, and psychological distress being the most reported issues. A dose-dependent relationship between caries severity (dmf index) and worse OHRQoL scores further emphasizes the cumulative burden of untreated disease. Younger parental age and maternal employment emerged as predictors of poorer OHRQoL, while child age and gender showed no significant association with ECOHIS scores. These results emphasize the necessity of parents’ education and early, focused preventive interventions to lessen the multifaceted burden of ECC in preschool-aged populations.

### Practice and policy implications

The following implications are proposed to link the findings to practice:

1. Implications for Public Health Policy:
  Oral Health in National Pediatric Care Programs: It involves the addition of oral health risk screening, non-cariogenic dietary counseling, and supervised tooth-brushing demonstrations and preventive practices in primary care clinics.
  Initiation of Targeted Public Awareness Campaigns: In addition to education of caregivers regarding the prevention and etiology of ECC, the campaigns should stress the psychosocial, developmental, and functional role of the primary dentition.
  Expanding Access to Reasonably Priced Health Care: Public health policymakers must consider programs providing funding assistance for low-cost preventive services, such as basic restorative services and application of fluoride varnish to high-risk children.
2. The Implications for Clinical Practice:
  Use of a Patient-Reported Outcome Measures (PROM) Framework: Regular use of the ECOHIS or other validated OHRQoL instruments in clinical care can promote shared decision-making, allow for full comprehension of the disease burden, and render treatment more personalized.
  Early and Aggressive Prevention: Specifically in children below the age of three, risk assessment for caries at the initial visit needs to be given high priority, and individualized prevention plans, such as high frequency of application of fluoride varnish, oral hygiene advice, and dietary counseling, need to be delivered.
  Support and Communication for Vulnerable Groups: Beneficial, supportive, and reassuring counseling can reduce distress and guilt among these groups and increase compliance with preventive guidance.

### Prospects

Longitudinal study designs need to be employed in future work in order to determine the effect of interventions and the causal influence between ECC and quality of life. To improve knowledge of variability in outcomes, studies must investigate unmeasured variables like parental oral health literacy, dental fear, and economic constraints.

## Supplementary Information

Supplementary Material 1.

Supplementary Material 2.

## Data Availability

Research data supporting this publication is available from the corresponding author upon request.

## Abbreviations

ECC: Early Childhood Caries
S-ECC: Severe Early Childhood Caries
OHRQoL: Oral Health-Related Quality of Life
ECOHIS: Early Childhood Oral Health Impact Scale
dmft: Decayed, Missing, Filled Teeth
CIS: Child Impact Section (ECOHIS)
FIS: Family Impact Section (ECOHIS)
