# Supplementary material for: Oral health related quality of life in a group of Egyptian preschool children with early childhood caries: a cross-sectional study
Source: BMC Oral Health. 2026 Feb 9;26:387. doi: 10.1186/s12903-026-07655-6 (PMC12937530; doi:10.1186/s12903-026-07655-6)
Supplement: Supplementary file 2 — Supplementary Material 2. [file 12903_2026_7655_MOESM2_ESM.pdf]

OHRQoL ECOHIS

Study objective: To investigate the impact of early dental caries on oral health-related quality of life in preschool children and their parents.

The study will include 260 children aged 3 to 5 years.

* Indicates required question

Demographic Data

1. Guardian's name

____

2. Age

____

3. Occupation

____

4. Child's name

____

5. Child's Age

____

6. Gender

Mark only one oval.

- Female
- Male

7. Address

8. ECC

Mark only one oval.

- Yes
- No

dmf Index

Untitled title

9. d *

10. m

11. f

____

12. How often has your child had pain in the teeth, mouth or jaws? (Child symptoms domain)

Mark only one oval.

- Never 0
- 1 Hardly Ever
- 2 Occasionally
- 3 Often
- 4 Very often
- 5 Don't Know

13. How often has your child had difficulty drinking hot or cold beverages?

Mark only one oval.

- Never 0
- 1 Hardly Ever
- 2 Occasionally
- 3 Often
- 4 Very often
- 5 Don't Know

14. How often has your child had difficulty eating some foods ?

Mark only one oval.

- Never 0
- 1 Hardly Ever
- 2 Occasionally
- 3 Often
- 4 Very often
- 5 Don't Know

15. How often has your child had difficulty pronouncing any words?

Mark only one oval.

- Never 0
- 1 Hardly Ever
- 2 Occasionally
- 3 Often
- 4 Very often
- 5 Don't Know

16. How often has your child missed preschool, daycare or school?

Mark only one oval.

- Never 0
- 1 Hardly Ever
- 2 Occasionally
- 3 Often
- 4 Very often
- 5 Don't Know

17. How often has your child had trouble sleeping?

Mark only one oval.

- Never 0
- 1 Hardly Ever
- 2 Occasionally
- 3 Often
- 4 Very often
- 5 Don't Know

18. How often has your child been irritable or frustrated?

Mark only one oval.

- Never 0
- 1 Hardly Ever
- 2 Occasionally
- 3 Often
- 4 Very often
- 5 Don't Know

19. How often has your child avoided smiling or laughing when around other children?

Mark only one oval.

- Never 0
- 1 Hardly Ever
- 2 Occasionally
- 3 Often
- 4 Very often
- 5 Don't Know

20. How often has your child avoided talking with other children?

Mark only one oval.

- Never 0
- 1 Hardly Ever
- 2 Occasionally
- 3 Often
- 4 Very often
- 5 Don't Know

21. How often have you or another family member been upset because of your child's dental
problems or treatments ?

Mark only one oval.

- Never 0
- 1 Hardly Ever
- 2 Occasionally
- 3 Often
- 4 Very often
- 5 Don't Know

22. How often have you or another family member felt guilty because of your child's dental
problems or treatments ?

Mark only one oval.

- Never 0
- 1 Hardly Ever
- 2 Occasionally
- 3 Often
- 4 Very often
- 5 Don't Know

23. How often have you taken time off from work because of your child's dental problems or dental treatments?

Mark only one oval.

- Never 0
- 1 Hardly Ever
- 2 Occasionally
- 3 Often
- 4 Very often
- 5 Don't Know

24. how often has your child had dental problems or dental treatments that had a financial impact on your family?

Mark only one oval.

- Never 0
- 1 Hardly Ever
- 2 Occasionally
- 3 Often
- 4 Very often
- 5 Don't Know

25. Guardian's Signature

____

This content is neither created nor endorsed by Google.

Google Forms
